# Prevalence of vitamin D deficiency and its associated risk factors among rural population of the northern part of the Persian Gulf

**DOI:** 10.1186/s12902-021-00877-5

**Published:** 2021-11-03

**Authors:** Maryam Marzban, Mohammadreza Kalantarhormozi, Mehdi Mahmudpour, Afshin Ostovar, Saeed Keshmiri, Amir Hossein Darabi, Abdolmohammad Khajeian, Amirreza Bolkheir, Azam Amini, Iraj Nabipour

**Affiliations:** 1 The Persian Gulf Tropical Medicine Research Center, The Persian Gulf Biomedical Sciences Research Institute, Bushehr University of Medical Sciences, Bushehr, Iran; 2 Department of Endocrine and Metabolic Disease, the Persian Gulf Tropical Medicine Research Center, Bushehr University of Medical Sciences, Bushehr, Iran; 3 Osteoporosis Research Center, Endocrinology and Metabolism Clinical Sciences Institute, Tehran University of Medical Sciences, Tehran, Iran; 4 Faculty of Medicine, Bushehr University of Medical Sciences, Bushehr, Iran; 5 Faculty of health, Bushehr University of Medical Sciences, Bushehr, Iran; 6 Department of Internal Medicine, Division of Rheumatology, School of Medicine, Bushehr University of Medical Sciences, Bushehr, Iran

**Keywords:** Vitamin D deficiency, Prevalence, Rural, Enrichment, Iran

## Abstract

**Background:**

Accumulating evidence indicates that vitamin D deficiency has been increased globally over the last two decades. However, the majority of these studies are concerned with cities and there is scant information regarding the prevalence of vitamin D in rural areas. The main aim of this study was to investigate the prevalence of vitamin D deficiency and its associated risk factors among the rural population in Bushehr province which shares the longest border with the Persian Gulf.

**Methods:**

The rural inhabitants of more than 25 years old from three mountainous, plain, and seashore areas of Bushehr province were selected through a stratified multi-cluster random sampling method. After obtaining the participants’ demographic and anthropometric data and their past medical history, serum 25-hydroxyvitamin D [25(OH)D] was measured using ELISA.

**Results:**

A total of 1806 (means ±SD, 46± 14years old) rural subjects (35 % males and 65 % females) participated in this study. The prevalence of vitamin D deficiency, insufficiency, and sufficiency were 28 %, 50 %, and 22 %, respectively. The deficiency of vitamin D in women was higher than in men (OR=1.27, 95 % CI: 1.05 to 1.54, *P*=0.04). There was a positive significant correlation between age and serum vitamin D levels. Men with vitamin D deficiency had higher BMI (*P*=0.008); this association was not observed among women (*P*=0.7). There was no significant difference between the food item’s consumption frequencies, and vitamin D status (*P*>0.05). The mountainous, and plain areas had the highest and lowest vitamin D levels, respectively.

**Conclusions:**

Although, Bushehr province is located in a sunny part of Iran, the prevalence of vitamin D deficiency was high among its rural population. The shift of their lifestyle patterns and rapid industrialization in these rural areas may be responsible. Therefore, the enrichment of dietary sources with vitamin D and the use of vitamin D supplements are recommended to tackle the high prevalence of vitamin D deficiency in the rural population of the northern part of the Persian Gulf.

## Introduction

The deficiency of vitamin D has been reported from all parts of the world, and there is a significant association between low serum levels of vitamin D and communicable and non-communicable diseases [1]. Accumulating evidence has shown that vitamin D presents beneficial effects on our tissues and organs.

Besides this, the more vitamin D concentration means the less occurrence of cancer and the related mortalities. In addition, vitamin D deficiency has significant association with high blood pressure, type I diabetes, multiple sclerosis, rheumatoid arthritis, and other autoimmune diseases [2].

Vitamin D deficiency has been defined at cut point serum levels of less than 20 ng/ml because at this level, parathyroid hormone begins to increase. Hence, this cut point is considered the physiologic definition for vitamin D deficiency [3]. However, the results of health outcome assessment of different studies indicated that all-cause mortality [4], cardiovascular diseases [5–7] breast and colorectal cancers [8, 9], diabetes mellitus [10, 11] acute respiratory tract infections [12] and SARS-CoV-2 positivity [13] appeared beyond the current target 25(OH)D concentration of 30 ng/mL (75nmol/L) for vitamin D deficiency. It is interesting that some of the aforementioned outcomes continued to improve up to 25(OH)D levels of 60-80 ng/ml.

Insufficient sunlight exposure, age- related decrease in vitamin D synthesis in skin, and low dietary intake are attributing factors for circulating levels of this vitamin. Therefore, vitamin D supplementation and sufficient sunlight exposure can protect most of the people from vitamin D deficiency. Five to ten minutes of direct sunlight exposure on the arms and legs between 10:00 AM to 3:00 PM during spring, summer, and autumn can prevent vitamin D insufficiency [14].

Because of the significant effect of vitamin D on bone mineral homeostasis, bone mineral density, and finally pick bone mass, several large multi-central interventional studies with vitamin D supplementation are under investigations in all over the world [15].

Even though there is a limited amount of vitamin D in plants, a sufficient amount of vitamin D3 could be found in fish oil, calf of liver, cheese, and eggs yolk [16]. In addition, acute liver failure, nephrotic syndrome, renal disease, rickets and hyperparathyroidism could be considered as etiologies for vitamin D insufficiency [6]. The geographical location, methods of skin coverage, skin color, and consumption of foods with lack of vitamin D are prominent causes for vitamin D insufficiency [16].

In a survey study which was done on American population from 2001 to 2006 (National Health, and Nutrition Examination Survey), it was shown that 32 % of participants had serum vitamin D level less than 20 ng/ml [17]. The studies in developing countries have shown that osteoporosis, increased risk of falling, and bone fracture were associated with an increase in the prevalence of vitamin D deficiency [18].

Moreover, the past two decades studies elucidated a high prevalence of vitamin D deficiency in China, Turkey, Saudi Arabia, and Iran which varied between 30 to 93 % [19].

Vitamin D deficiency was reported in 75.1 % of females and 72.1 % of males from the Iranian Multi-Centric Osteoporosis Studies (IMOS). The result of this study was consistent with those results that had been reported with other studies from the Middle East region. [20]. A comprehensive epidemiological and ecological descriptive study on vitamin D status in Iran showed that the mean of vitamin D concentration was 25.4 ng/ml which was in the range of vitamin D insufficiency [21].

The high prevalence of vitamin D deficiency among the Iranian population may be due to the shift of Iranian lifestyle patterns towards the urbanization and industrialized lifestyles resulting in low sunlight exposure that accentuates the low dietary intake of vitamin D in this country [20]. In addition, air pollution is an important affecting factor for vitamin D deficiency in the large cities [22]. Although similar prevalence rates for vitamin D deficiency in urban and rural areas of Asia have been reported, in some studies, the urban subjects in comparison to the rural subjects had more prevalence of vitamin D deficiency [15].

In Iran, the majority of studies have been done in urban areas and capital cities [19, 23, 24]. There is a lack of information about the prevalence of vitamin D deficiency and its serum levels in rural areas [19].

Therefore, the limited information about vitamin D in rural areas may prevent to design strategies to combat vitamin D deficiency in Iranian rural subjects who are rapidly changing their lifestyle towards urbanization and industrialization. Hence, this phenomenon may accelerate the situation of vitamin D deficiency among this rural population. The aim of this study was to investigate the prevalence of vitamin D deficiency and its associated risk factors among rural population in Bushehr province which has the longest border with the Persian Gulf.

## Materials and methods

### Community sampling

Bushehr province which is located in the northern part of the Persian Gulf, was divided in three geographical (the northern, the central, and the southern) parts. Each geographical part was then divided into three mountainous, plain, and seashore areas in order to assess the effect of latitude, dietary habits, and socio-economic status of inhabitants on their serum vitamin D levels. The sample size of each part was proportional to the size of each population. The household was selected as the sampling unit which were randomly selected systematically using national census data.

A total of 1806 rural inhabitants with ≥ 25 years old were selected. The exclusion criteria included being non-native, inability to give blood sample, disability to answer the questions, and being less than 25 years old. The participants completed a questionnaire including their anthropometric, demographic data, past medical history, gravidity, menopausal status, nutritional conditions and supplementary consumptions as well as their drug history.

### Examinations

Examination were conducted in the primary health care centers attached to Bushehr University of Medical Science (BUMS). Height, and weight were measured using stadium meter. Heavy outer garments, and shoes were removed before measuring heights, and weights. Body mass index (BMI) was calculated. Waist circumference was defining at the mid waist level between the costal margin and iliac crest. Hip circumference was measured at the greater trochanters. A 3 milliliter (ml) sample of blood was taken from all participants, and all samples were promptly centrifuged, and sera were separated, and kept frozen at -80 until they were used.

Serum vitamin D levels were measured using a commercial ELISA kit Immunodiagnostic Systems Limited (The Boldons, United Kingdom). The reportable range of the assay was 6.5-100 ng/ml. Its limit of detection was 2.7 ng/ml. The range of inter-assay %CV of the kit was 1.9 to 3.7 ng/ml.

Serum 25(OH)D levels were defined sufficient if they were between 30 and 100 ng/ml, insufficient if varied between 20- <30 ng/ml, and deficient at levels of <20 ng/ml.

### Statistics

The statistical analysis was performed using STATA version 14 (StataCorp Pty Ltd, College Station, TX, USA). The Kolmogorov-Smirnov test (KS-test) was used to determine the normality of data distribution. For descriptive data, frequency, mean, median, and standard deviation were used. The statistical significance of variables between groups was analyzed using X^2^ test. A univariate and multivariate analysis was performed to evaluate the association between 25(OH)D (dependent variables) and other co-factors including (age, sex, marital status, anthropometrics data, sun-light exposure, sun protection factor (SPF), dietary, and supplementary consumption).

Multiple logistic regression analysis was used to ascertain the association between vitamin D deficiency status and its associated risk factors. *P*<0.05 was considered statistically significant.

## Results

A total of 1806 rural subjects including 631 (35.0 %) men and 11,75 (65.0 %) women, participated in the study. The characteristics of the participants were shown in Table 1. The subject ages ranged from 23 to 94 years old (mean ± standard deviation 46± 14 years). The prevalence of vitamin D deficiency and insufficiency were 28 % (505 subjects), 50 % (913 subjects), respectively. A total of 394 subjects (22 %) had sufficient serum vitamin D levels.

**Table 1:** The demographic and anthropometric data of the rural population in the northern part of the Persian Gulf

Variable	Number (%)	Variable	Number (%)
Age		Working outside the room	596 (33.0)
<22	12 (1.0)	The mean of sun exposure (hours per week)	
22-39	653 (36.0)	< 1 h	1241 (71.0)
40-59	743 (41.0)	2-10 h	138 (8.0)
>60	403 (22.0)	11-20 h	135 (8.0)
Sex		>21 h	239 (14.0)
Male	631(35.0)	The sun exposure per week	
Female	1175 (65.0)	< 5 min	109 (6.0)
Marital status		5-15 min	347 (19.0)
Single	184 (10.0)	16-30 min	370 (20.0)
Married	1478 (82.0)	>30 min	875 (48.0)
Widow	120 (67.0)	Consumption of SPF*	280 (15.0)
Separated	22 (1.0)	Waist circumference (men)	91 (5.0)
BMI**		Waist circumference (women)	675 (37.0)
Normal	41 (2.0)	WHR***	
Over weight	690 (38.0)	<0.9 for men	438 (24.0)
Obese	106 (59.0)	<0.85 for women	870 (48.0)

* SPF: Sun Protection Factor

**: BMI: Body Mass Index

*** WHR: Waist to Hip Ratio

The deficiency of vitamin D in women was higher than men (OR=1.27 95 % CI: 1.05 to 1.54, *P*=0.04). Furthermore, 537 (29 %) of the participants (22 % of women, and 42 % of men) had sun exposure more than 15 min per-day 2 to 3 times per week. There was a significant difference for mean sun-light exposure among sufficient (0.82±1.22 h per day), insufficient (0.67±1.10 h per day) and deficient (0.54±1.02 h per day) groups of vitamin D (β = 0.64 [0.11-1.17], *p*=0.016; Table 1).

The prevalence of consumption of oral calcium, vitamin D, calcium + vitamin D, and parenteral vitamin D were 37 (2 %), 239 (13 %), 32 (2 %), 7 (0.4 %), respectively. The negative consumption of tuna and curd was found for a half of the participants, while 72 % of them reported positive consumption of egg. Also, more than 50 % of the participants reported more than the median consumption of dough and milk. Furthermore, 148 (29 %) and 327 (19 %) of the participants reported to have history of hypertension and diabetes mellitus, respectively.

There was also a significant association between age and serum vitamin D levels (P≤0.001).

The rural subjects between 30 and 39 years old and elderly participants of the study with more than 80 years old had the highest and lowest amounts of vitamin D deficiency, respectively.

In men, their serum levels of vitamin D were increased with increasing their age, while by increasing their height, weight, and BMI, it was decreased. However, in women, only age had a positive association with vitamin D serum levels. In addition, men with vitamin D deficiency had higher BMI (*P*=0.008); this association was not observed among women (*P*=0.74). No association could be found between waist to hip ratio in relation to the status of vitamin D levels (*P*>0.05) (Table 2). In addition, there was no significant difference between the food items consumption frequencies, and vitamin D status (*P*>0.05) (Table 3).

**Table 2:** The association among age, anthropometric indices and vitamin D status among rural population of the northern part of the Persian Gulf

	Vitamin D deficient*	Vitamin D insufficient**	Vitamin D sufficient***	Crude β	P-Value	Vitamin D deficient	Vitamin D insufficient	Vitamin D sufficient	Crude β	P-Value
	**Male**					**Female**				
Age	46. ± 15	47. ± 14.	50± 16.	0.05 (0.00 to 0.11)	0.043	44. ± 13.	47± 14.	48± 14.	0.11 (0.06 to 0.16)	0.0001
Height	170. ± 7.	170± 7.3	170± 7.	-0.11 (-0.02 to 0.00)	0.048	160 ± 11.	160± 14.	160± 8.	0.00 (-0.05 to 0.06)	0. 86
Weight	76 ± 15.	73. ± 14	70.± 11.	-0.09 (-0.15 to -0.03)	0.002	67± 14	68 ± 14.	66. ± 14.	-0.03 (-0.09 to 0.01)	0.13
Body mass index	26 ± 4.7	25. ± 4.3	25 ± 3.4	-0.25(-0.45 to -0.06)	0.01	27. ± 5.4	28. ± 5.7	27.± 6.0	-0.08 (-0.21 to 0.04)	0.19
Waist circumference	91. ± 17.	90 ± 17	89 ±14.	-0.02 (-0.07 to 0.03)	0.42	90. ± 17	91 ± 17.	89 ± 20	-0.02 (-0.06 to 0.01)	0.29
Hip circumference	98 ± 17.	97 ± 17.	95 ± 15.	-0.03 (-0.08 to 0.01)	0.19	100. ± 18	100 ± 20.	99. ± 21	0.09 (-0.04 to 0.02)	0.60

* Serum 25(OH)D levels of <20 ng/ml

**Serum 25(OH)D levels between 20- <30 ng/ml

*** Serum 25(OH)D levels between 30-100 ng/ml

**Table 3:** The nutritional consumption of food items across vitamin D groups among rural population of the northern part of the Persian Gulf

Variable	Vitamin D deficient (<20 ng/ml)	Vitamin D insufficient (20- <30 ng/ml)	Vitamin D sufficient (30-100 ng/ml)	P value
Egg consumption (grams per day)	24. ± 19.	24. ± 32.	23±19	0.39
Fish consumption (grams per day)	25.± 22	23. ± 20.	23. ± 20.	0.09
Tuna consumption (grams per day)	1.2 ± 3.7	1.1 ± 3.2	1.00 ± 3.2	0.48
Milk consumption (grams per day)	76. ± 95.	80.± 120	76. ± 100	0.79
Cheese consumption (grams per day)	18. ± 18.	18 .± 17.	17. ± 20.	0.92
Dough consumption (grams per day)	130 ± 110	130 ± 110	130± 130	0.95
Curd consumption (grams per day)	3.2 ± 6.4	3 0.0± 9.0	3. ± 10	0.80

In multivariate analysis, sun exposure more than 15 min per-day 2 to 3 times per week (OR=0.93, 95 % CI: 0.88-0.99; *p*=0.032) and BMI (OR=1.04, 95 % CI:1.04, 95 % CI:1.0-1.08; *p*=0.017) in men, and age (OR=0.98 95 % CI: 0.97-0.99, *p*=0.0001) in women had significant association with vitamin deficiency status (Table 4).

**Table 4:** Multivariate adjusted odds ratios (OR) and 95 % confidence intervals relating vitamin D deficiency status and its associated risk factors among rural population of the northern part of the Persian Gulf

	Vitamin D deficiency			Vitamin D deficiency		
	Male			Female		
Variable	OR	95 % CI	P-Value	OR	95 % CI	P-Value
Age	0.99	0.97 to 1.00	0.096	0.98	0.97 to 0.99	0.0001
sun exposure more than 15 min per-day 2 to 3 times per week	0.93	0.88 to 0.99	0.032	0.95	0.90 to 1.00	0.059
Taking vitamin D supplements	0.84	0.53 to 1.33	0.481	1.09	0.81 to 1.47	0.539
Marital Status	1.22	0.77 to 1.94	0.389	0.96	0.76 to 1.20	0.733
Body Mass Index	1.04	1.00 to 1.08	0.017	0.99	0.97 to 1.01	0.570

There was a significant difference between the score of gravidity and vitamin D status (*P*<0. 001). However, there was no significant association between the months of breast feeding, and serum vitamin D levels (*P*=0.2). However, 292(46 %) of subjects with vitamin D deficiency, 196 (31 %) of subjects with vitamin D insufficieny, and 143(22 %) of subjects with sufficient serum vitamin D  levels reported that they had history of breast feeding; however, no significant differences could be found among them (*P*=0.08).

**Fig. 1:**
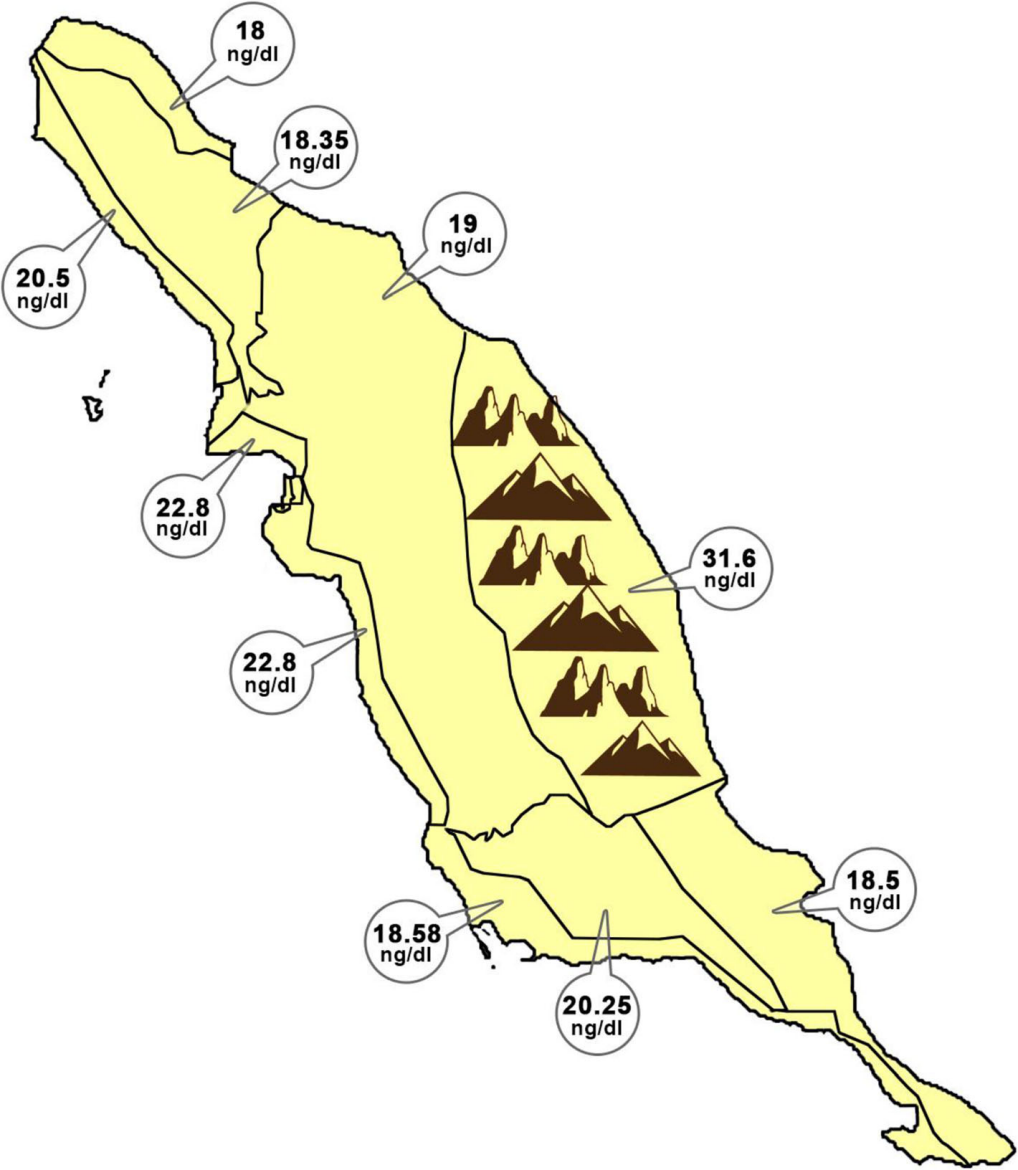
The age-adjusted medians of serum vitamin D levels in the mountainous and plain regions of the rural areas of the northern part of the Persian Gulf

The mountainous, and plain regions of the rural areas had the highest, and lowest vitamin D levels, respectively (Fig. 1).

## Discussion

In this population-based study which was done in all rural districts of Bushehr province, it was shown that a half of the rural participants had vitamin D deficiency, more than one to five of the participants had vitamin D insufficiency, and one to five of total population had sufficient vitamin D levels.

Unfortunately, there are limited studies on rural population in Iran regarding vitamin D levels, and most of the studies have been done on the capital cities; therefore, it is impossible to compare vitamin D serum levels of rural and urban populations. The only study that evaluated the Iranian rural and urban populations was done on Guilan province which compared 750 postmenopausal women in rural and urban areas and demonstrated that vitamin D deficiency was more common in urban than rural subjects [25]. This difference among rural and urban subjects was also observed in other countries [26]. In a systematic review and meta-analysis study in Africa it was shown that the mean of serum vitamin D levels in urban places was less than the rural areas [27]. This difference could be explained with different lifestyles, jobs, and habitual conditions because the rural inhabitants live more outdoor and expose to more sun-light, resulting with more absorption of vitamin D by their skin; in another aspect, the urban lifestyle patterns cause less amount of vitamin D absorption via sun-light exposure or less amount of dietary vitamin D intake through food habits [25, 27].

Although, in the current study, we could not compare the prevalence of vitamin D deficiency in urban and rural regions, we found that the prevalence of vitamin D deficiency in rural areas was at least similar to those prevalence rates that has been reported from Bushehr city (the capital of Bushehr province), the middle east and south east of Asia, and China [19, 28–30]. In two systematic reviews and meta-analyses from Iran, it was reported that more than a half of the Iranian population, especially those who live in capital cities, had vitamin D deficiency [23, 24].

In Iran, the fundamental infrastructural and economical changes during the post 1979 revolution produced a trend in rural areas to adopt the behaviors, values, attitudes, and traditions of urbanism. This transformation created a change of lifestyle from rural to urban patterns including consumerism, employment in non-agricultural jobs and activities [31].

The transformation of people’s lifestyles along with changes in living conditions and settlement models from rural to urban promoted an unhealthy lifestyle in the northern Persian Gulf region. We assessed prevalence of unhealthy lifestyles in 3723 participants aged > or = 25 years in this region; over 60 % had unhealthy body weight, only 8.3 % ate the recommended amount of fruits and vegetables, 70.6 % were physically inactive [32]. Therefore, the adoption of an unhealthier lifestyle accompanied with more indoor activities and lower sun exposure by the rural population of the northern Persian Gulf region may have induced their mean vitamin D serum levels to approach to their urban counterparts.

In our study, we found a linear relationship between serum vitamin D levels and sun-light exposure. In the European population, it has been revealed that sun exposure of 18 % of body surface area for 15 min per-day, 2 to 3 times per week is sufficient for absorbing vitamin D [33]. However, in the current study, only 29 % of participant had fulfilled the above criteria. This low level of sun-exposure in the rural areas may be due to the mentioned changes of lifestyle towards the urban patterns. The cultural factors may also have an effect on the amount of villager’s sun-light exposure because of the types of their clothing which cover their arms and legs for all seasons.

The effect of body coverage on circulating vitamin D is so important that in sunny countries such as Saudi Arabia and the United Arab Emirates, a high prevalence of vitamin D deficiency could be found; likewise, in Iranian sunny cities such as Zahedan and Isfahan, a high vitamin D deficiency have been reported [34–37].

In another aspect, the effect of sun exposure on vitamin D serum levels could be ascribed by its seasonal patterns of sun-light exposure; in winter, we can expect to obtain less amount of vitamin D by decreasing in sun-light exposure [37]. In the current study, all the serum samples were obtained during winter; therefore, the effect of seasonal patterns could not be evaluated. However, it seems that there is a complex interaction between the effect of season and bio-cultural factors.

Surprising, the mean serum vitamin D was highest in winter and lowest in the summer in a sunny country like the United Arab Emirates [34]. The climate of the United Arab Emirate is very similar to Bushehr province in the northern parts of the Persian Gulf. It could be postulated that the high temperature of these places induces people live most of their times indoor to escape the hot condition during summer times. Hence, they receive less amounts of sun-light exposure, leading to the lowest range of serum vitamin D levels during summer. In order to elucidate the complex interaction of cultural, and bio-environmental factors on circulate vitamin D levels, more studies during different seasons are warranted.

In this study, an association between vitamin D serum levels and age was found. It was interesting that the minimum serum levels of vitamin D were observed in 30 to 39 years old age group; and surprisingly the highest levels of vitamin D were found among rural subjects who had more than 80 years old. In an Iranian meta-analysis the highest prevalence for vitamin D deficiency was found among the 20 to 50 years old age group [24]. In contrast, in a previous study, children and elder people had the highest prevalence of vitamin D deficiency [38]. In Iran, due to not including older people in the previous population based studies about the prevalence of vitamin D deficiency, a comparison would not be possible [23].

The consumption of multivitamins and vitamin D pills by the elderly may lead to a lower prevalence of vitamin D in this age group in comparison to the younger participant in our study. Another contributing factor for this difference may be the changes of life styles of younger people that induce them to choose living and working indoor places with resulting in less sun-light exposure. In consistent with our finding, a positive correlation between age and vitamin D serum levels was found in Zahedan city (the capital of Sistan, and Balochestan province in the south east of Iran) [23, 24]. Likewise, the younger age group had a higher prevalence of vitamin D deficiency than older age group in Isfahan city ( the capital of Isfahan province in the center of Iran) [37]. Taken together, the change of lifestyle patterns and the trend of younger people to stay in indoor places, and living in apartments may explain this difference for prevalence of vitamin D deficiency among different age groups.

In our study, the prevalence of vitamin D deficiency was more common in men than women. The results of two meta-analyses studies from Iran showed that the prevalence of vitamin D deficiency, like other Asian countries, was more common in women than men [23, 24]. In a trend prevalence study of vitamin D deficiency during 1990-2010 in Iran, it was also reported that women gained a higher vitamin D deficiency than men year-over-year[39].

There are contradictory results about vitamin D deficiency in relation to sex in the world. In contrast to a previous study in America, no difference could be found between sex groups in relation to vitamin D deficiency in NHANES 2001-2004 [40]; but, in a later study in America, women had a higher vitamin D levels than men [41]. In Africa, the prevalence of vitamin D deficiency was higher in women than men [27]. In the United Arab Emirates (UAE), the prevalence of vitamin D deficiency was similar in both sexes [42]. It has been suggested that cultural and religious factors might be the causative factors for the observed higher prevalence of vitamin D among Muslims women; for instance, in Lebanon, the Muslim women had lower vitamin D serum levels than the Christian women [43]. These contradictory results indicate that other contributing factors beyond veiling should be considered to explain this difference.

Obesity is another condition that has a connection both to the patterns of life style and vitamin D serum levels [44]. In many epidemiological studies from tropical countries, a negative significant association between obesity and serum 25(OH)D levels were reported after controlling for potential confounders [45–47]. According to the results of a meta-analysis, that investigated relationship between obesity and vitamin D deficiency, a higher prevalence of vitamin D deficiency was found among obese subjects independent to traditional risk factors including the Human Development Index of the study location [48]. Interestingly, in another systematic review and meta-analysis of randomized controlled trials, it was reported that obesity in adults attenuated the effect of vitamin D supplementation [49]. The body fat content has a reverse correlation with vitamin D concentration. This inverse correlation may be due to the decrease bio-availability of vitamin D3 from dietary sources and skin because of the deposition of vitamin D in body fat compartments [50]; even the elder subjects with high body fat and higher body mass index have lower levels of 25(OH)D levels [51]. Other factors contribute to the inverse relationship between body mass index and vitamin D levels, such as decreased mobility in obese people, which reduces their exposure to sunlight [44].

In our study, men with vitamin D deficiency had a higher anthropometric index (BMI) than men with sufficient vitamin D levels, while no difference was observed between the two groups in women; therefore, other contributing factors beyond obesity should be considered that modify the effect of obesity, such as intake of vitamin D supplements and the number of pregnancies.

Although the current study is one of the largest studies that investigated vitamin D deficiency in the Iranian rural subjects, it has some limitations. One of these limitations is non-repetition of measurements for vitamin D levels during different seasons. The physical activity, and their smoking status of the participants were not also assessed. However, the evaluation of nutritional status as well as sun exposure which were addressed in this study could be ascribed as one of its strengths.

Another strength of the study is its design so that the effect of the latitude (mountainous, plain and coastal places) of living areas on the level of vitamin D could be evaluated. In this study, the northern half part of the rural mountainous area of Bushehr province had a higher mean of vitamin D level than the plains and coastal areas. There was no significant difference in daily consumption of dairy, and milk products between mountainous area, and other parts of this province. This difference may be partly originated from the altitude effect (AE) on solar radiation intensity because UV B photons undergo less scattering and absorption by traversing the thinner atmosphere of the mountainous areas. The resulting effect will be sufficient exposure to UV B for synthesis of more vitamin D in the epidermis of the mountain dwellers than the plain dwellers. Although we did not assess the level of air pollution in different geographical areas in this study, it has been shown that air pollution levels including many organic aerosols have a significant reduction in UV spectral irradiances [52–54].

By considering widespread vitamin deficiency in all age groups and that natural sources of vitamin D cannot maintain circulating 25(OH)D levels in the recommended target of 30-50 ng/ml (75-125 nmol/L), systemic vitamin D food fortification and targeted vitamin D supplementation may be suggested as effective strategies in order to combat with vitamin D deficiency [55, 56]. These strategies have been considered in Asian countries [15], US, Canada and Finland [57]. In India, the programs of enrichment of a dietary source with vitamin D, supplementation of vitamin D plus calcium, and inclusion of local fortified food items were launched by local governmental agencies, and other state holders [58]. It has been shown in a modelling study that fortifying at least one food vehicle (edible plant-based oil, wheat flour and milk) can increase per capita vitamin D supply to maintain serum 25(OH)D≥ 25 nmol/L in low/lower-middle income countries [59]. An individual-level simulation model revealed cost effectiveness of the combined strategy of wheat flour fortification and targeted vitamin D supplementation [60].

In Iran, a community-based interventional trial of vitamin D fortified milk has been started [61]. The results of this trial could be applied in different parts of the country to tackle vitamin D deficiency in the near future. However, it is important  to incorporate coverage for processing, storage, and cooking in fortification processes . Otherwise, the effectiveness of fortification may be diminished considerably, particularly in the case of less stable vitamins. Therefore, considering multiple fortification vehicles for delivery of the same and different nutrients is prudent, as it may increase the overall effectiveness of fortification and allow natural variation in population dietary patterns to be exploited for more effective targeting of intake deficiencies in different subgroups [62].

The supplementation of vitamin D has been suggested as a safe and effective way to tackle vitamin D deficiency [55]. The intake of vitamin D supplements were reported to be low in Iran [23, 63]. In the current study, only 16 % of participants reported taking vitamin D or calcium plus vitamin D supplements. Since vitamin D is crucial for preventing of several chronic, communicable and non-communicable diseases[55], the development of vitamin D supplementation should be considered as an national need to meet its requirements.

## Conclusions

Although, Bushehr province is located in a sunny part of Iran, our study showed that there is a high prevalence of vitamin D deficiency among the rural population of Bushehr province. This prevalence is similar to those prevalence rates that have been reported from the urban areas of Iran. This similarity of prevalence may be partly due to the shift of the rural dwellers life styles patterns that originated from the rapid industrialization development in these rural areas.

## Data Availability

The datasets used and/or analyzed during the current study are available from the corresponding author on reasonable request.

## Abbreviations

BMI: Body mass index
ELISA: enzyme-linked immunosorbent assay
IMOS: Iranian Multi-Centric Osteoporosis Studies
BUMS: Bushehr University of Medical Science
UK: United Kingdom
KS-test: The Kolmogorov-Smirnov test
SPF: sun protection factor
NHANES: National Health and Nutrition Examination Survey
UAE: United Arab Emirates
